# Suppression of p66Shc prevents hyperandrogenism-induced ovarian oxidative stress and fibrosis

**DOI:** 10.1186/s12967-020-02249-4

**Published:** 2020-02-17

**Authors:** Daojuan Wang, Tingyu Wang, Rong Wang, Xinlin Zhang, Lei Wang, Zou Xiang, Lingjia Zhuang, Shanmei Shen, Hongwei Wang, Qian Gao, Yong Wang

**Affiliations:** 1 State Key Laboratory of Analytacal Chemistry for Life Science & Jiangsu Key Laboratory of Molecular Medicine, Medical School, Nanjing University, Nanjing, 210093 China; 2 Department of Cardiology, Affiliated Drum Tower Hospital, Nanjing University School of Medicine, 321 Zhongshan Road, 210008 Nanjing, Jiangsu Province China; 3 Department of Health Technology and Informatics, Faculty of Health and Social Sciences, The Hong Kong Polytechnic University, Hung Hom, Kowloon, Hong Kong, China; 4 Department of Endocrinology, The Affiliated Drum Tower Hospital, Medical School, Nanjing University, Nanjing, 210093 China

**Keywords:** PCOS, Granulosa cells, p66Shc, Fibrotic factors, Reactive oxygen species

## Abstract

**Background:**

Rats with hyperandrogen-induced polycystic ovary syndrome (PCOS) have been shown to develop ovarian oxidative stress (OS) and fibrosis. The Sirt1 agonist, resveratrol, can reduce OS through inhibiting p66Shc in other models of OS.

**Methods:**

We created a rat PCOS model with increased OS levels following treatment with one of the two androgens, dehydroepiandrosterone (DHEA) and dihydrotestosterone (DHT). The PCOS related features were determined by measurement of malondialdehyde (MDA) and superoxide dismutase (SOD) levels or by examining the reactive oxygen species (ROS) levels using the DCF-DA probe. The potential mechanisms by which p66Shc/Sirt1 mediates ovarian fibrosis were explored by western blotting, quantitative reverse transcription-PCR, immunofluorescence staining, and immunohistochemistry.

**Results:**

Hyperandrogen dramatically augmented OS and activation of fibrotic factors in the ovary. Our data demonstrated that treatment with resveratrol enhanced Sirt1 and decreased ovarian OS as well as inhibited phosphorylation of p66Shc both in vivo and in vitro. The treatment suppressed fibrotic factor activation and improved ovarian morphology. Lentivirus- or siRNA-mediated p66Shc knockdown resulted in a dramatic enhancement of Sirt1 expression, down-regulation of ROS and suppression of fibrotic factors in granulosa cells. Moreover, p66Shc overexpression markedly increased the expression of fibrotic factors. Additionally, silencing Sirt1 induced a dramatic increase in p66Shc and enhanced activation of fibrotic factors.

**Conclusions:**

p66Shc may be a direct target of Sirt1 for inducing ROS and thus promoting fibrosis. Further exploration of the mechanisms of p66Shc in both fibrosis and OS may provide novel therapeutic strategies that will facilitate the improvement in PCOS symptoms and reproductive functions.

## Background

Polycystic ovary syndrome (PCOS) patients are characterized by androgen excess, insulin resistance, chronic anovulation and ovarian fibrosis, which are the most common causes of irregular menstruation, amenorrhea, infertility, hirsutism and acne in young women [1]. Androgens such as dehydroepiandrosterone (DHEA), testosterone and dihydrotestosterone (DHT) play important roles in the development of the ovary [2]. The physiological functions of the androgens are predominantly mediated by the activation of the androgen receptor (AR), which is a member of the steroid hormone receptor superfamily of ligand-activated transcription factors [3]. Androgen excess is considered as one of the most important factors contributing to PCOS [4, 5]. Evidence from clinical research has revealed increased levels of oxidative stress (OS) in PCOS patients [6]. Recent research demonstrated that androgen-induced PCOS rats demonstrated ovarian fibrosis which can compromise ovarian functions [7]. Studies in lungs and kidneys have shown that the pro-fibrosis factor transforming growth factor-beta (TGF-β) can stimulate the production of reactive oxygen species (ROS), which in turn activates fibrogenic factors such as TGF-β and connective tissue growth factor (CTGF) [8].

OS is involved in the pathophysiology of many diseases, including atherosclerosis, diabetes, and tumor formation as well as in aging [9]. OS is mainly caused by the imbalance between pro-oxidants and anti-oxidants, and the ratio can be regulated by the inactivation of ROS and reactive nitrogen species (RNS) and other antioxidant mechanisms [10, 11]. Mitochondria are major sites for the generation of superoxide radicals that can detoxify ROS. However, excess ROS generation can destroy these defense mechanisms and lead to mitochondrial damage [12]. Accumulating evidence suggests that common manifestations of PCOS, such as hyperandrogenism, obesity, insulin resistance, and abdominal hyperlipidemia, can result in partial or systemic OS, further exacerbating the patient’s metabolic abnormalities, including insulin resistance and hyperandrogenism, thus forming a vicious cycle [13, 14].

Resveratrol is a natural compound found in grapes, mulberries, peanuts, and red wine [15]. Resveratrol is known to promote antioxidant defenses by regulating a host of antioxidant enzymes. Recent studies have suggested that resveratrol is an agonist of sirtuin type 1 (Sirt1) and exerts a wide variety of beneficial effects in a Sirt1-dependent manner to prevent the development of many illnesses, through, e.g., antidiabetic, antioxidant and antiinflammatory approaches [16]. In addition, resveratrol has an important effect on ovarian development and oocyte apoptosis [17, 18]. However, the mechanisms by which resveratrol regulates Sirt1 to retard ovarian OS have not been fully elucidated.

The 66-kDa Src homology 2 domain-containing protein (p66Shc) belongs to the ShcA family of adaptor proteins, which plays an important role in the production of mitochondrial ROS [19]. In response to oxidative stimuli, p66Shc gets phosphorylated at serine 36 (S36) of the CH2 domain, and then isomerized, dephosphorylated and translocated to mitochondria, which in turn accelerates the production of ROS [20–22]. When cells are exposed to oxidative stimuli, p66Shc is deacetylated by Sirt1 lysine deacetylase, and Sirt1-mediated deacetylation of p66Shc reduces the production of ROS [23, 24]. P66Shc is a protein that regulates cell senescence and it can promote aging by inducing apoptosis and necrosis of many types of cells. Its absence enhances cell resistance to ROS and prolongs cell survival. Sirt1 and p66Shc have opposing effects on vascular functions [25]. Moreover, down-regulation of p66Shc leads to epigenetic up-regulation of Sirt1. However, whether p66Shc directly targets Sirt1 for lysine deacetylation and whether dynamic lysine acetylation of p66Shc governs its oxidative function are not known [26].

Both ovarian hyperfibrosis and increased OS are seen in PCOS patients. However, the relationship between the two has not been reported. Studies on smooth muscle cells and endothelial cells have shown that TGF-β increases the level of ROS and potentially reduces the production of antioxidants. On the other hand, ROS can activate TGF-β. Consistently, TGF-β production can be suppressed by antioxidants [8].

In this study, we aimed to investigate the possible inhibition of hyperandrogenic ovarian fibrosis by preventing p66Shc-induced OS. Because of the lack of specific antagonists of fibrosis, Sirt1 is employed to partially reduce the expression of p66Shc and thus exert an anti-OS activity. Recent studies have shown that PCOS is accompanied by low-grade chronic inflammation. Resveratrol may be able to suppress the proinflammatory activity and may be potent in treating metabolic disorders that usually are associated with PCOS [27]. Furthermore, experimental evidence has demonstrated a protective effect of resveratrol against several conditions mediated by OS [28]. Thus, we undertook to examine the effect of resveratrol on protection of PCOS rats from ovarian fibrosis mediated by p66Shc-induced OS.

## Materials and methods

### Animals and experiment protocol

Female Sprague–Dawley (SD) rats (21 days old, 50–60 g, n = 21) were purchased from Qinglongshan, Nanjing, China. For creating a PCOS model, rats received a daily hypodermic injection of DHEA (6 mg/100 (g·d), n = 7) for 35 consecutive days [29]. Naïve rats (n = 7) were used as controls. In addition to DHEA, some of the rats also received a daily injection of resveratrol (100 mg·kg^−1^·day^−1^, n = 7) throughout the DHEA treatment period. All rats were weighed once every other day. All animals were housed in a specific pathogen-free (SPF) environment (Jiangsu Key Laboratory of Molecular Medicine) with a temperature of 24 ± 1 °C, and a light/dark cycle of 12/12 h. Free access to food and water was provided. On day 36, all rats were killed, and both ovaries were harvested. Next, we removed fat around the ovary and weighed the ovaries. Their blood and other various tissues were harvested and immediately stored at − 80 °C for molecular analysis. The experiments were carried out in accordance with the principles and guidelines for the use of laboratory animals and approved by the institutional research animal committee of Nanjing University.

### Isolation and culture of granulosa cells (GC) and theca cells (TC)

For obtaining rat pre-ovulatory follicles, immature 23-day-old female rats were injected with pregnant mare serum gonadotropin (PMSG) (20 IU) to enhance multiple follicular development. After 48 h, ovaries were harvested for isolation of the GCs and TCs following anaesthesia with 5% chloral hydrate. Cell debris in the GCs was removed using 70-μm cell strainers, and debris in TCs was removed using 100-μm cell strainers [30, 31]. The primary GCs and TCs were cultured in Dulbecco’s modified Eagle’s medium/nutrient mixture F-12 (DMEM-F12) containing 10% fetal bovine serum (FBS, Wisent, Canada) and 1% penicillin–streptomycin solution (Gibco, USA) in 6-well (1 × 10^6^/well) or 12-well culture plates (1 × 10^5^/well), (adherent growth on the round coverslip), in a 5% CO_2_/air atmosphere and maintained at 37 °C.

### p66Shc knockdown by siRNA and siRNA-containing lentiviral vectors

The p66Shc small interfering RNA (siRNA) target sequence (5′-GCAAACAGAUCAUUGCCAATT-3′) and the control siRNA sequence (5′-TTCTCCGAACGTGTCACGT-3′) were designed at the GeneChem Company (GeneChem, Shanghai, China). The virus titer used was 1 × 10^9^ (TU/ml). Primary GCs were transfected with the siRNA (Keygen Biotech, China) using Lipofectamine 2000 (Invitrogen, USA), or a siRNA-containing lentiviral vector (GeneChem, Shanghai, China) using HitransG P with a 72-h incubation. Next, the cells were incubated with DHT (500 nM for 24 h), resveratrol (50 μM for 24 h), transforming growth factor-β1 (TGF-β1, 2 ng/ml for 24 h), or SOD (20 IU/ml for 24 h) for various assays.

### Plasmids

pCMV-Shc1-HA was designed and amplified at the GeneChem Company Company (GeneChem, Shanghai, China). Plasmids were transfected into GCs using Lipofectamine 2000 (Invitrogen, USA) with a 48-h incubation. Next, cells were incubated with TGF-β1 for various assays.

### Serum hormone measurement

Blood samples were collected from the superior vena cava. Serum was separated immediately and stored at − 80 °C for further determination of testosterone (T) and estradiol (E2) levels by an enzyme-linked immunosorbent assay (ELISA) (Elabscience Biotechnology, China).

### Measurement of malondialdehyde (MDA) and superoxide dismutase (SOD) levels

The ovaries were cut into pieces of about 15 mg, homogenized, followed by collection of the supernatant. The MDA and SOD levels in the ovarian tissue supernatant, serum and GCs were measured according to a previously described method using the Lipid Peroxidation MDA Assay Kit (Beyotime, China) and SOD Activity Assay Kit (Beyotime, China).

### Hematoxylin and eosin (H&E) staining

Paraffin slices were stained with hematoxylin and eosin in order to examine the pathological structural alterations of the rat ovary under an optical microscope (Leica Microsystems, Germany).

### Sirius Red and Masson staining

Slices of ovarian collagen were stained by sirius red and matson to reveal ovarian fibrosis in a hyperandrogenic environment and to demonstrate the inhibitory effects of resveratrol on fibrosis.

### Immunohistochemistry (IHC) and immunofluorescence (IF)

Samples were left to incubate overnight at 4 °C with specific antibodies against p66Shc (phosphor S36) (Abcam, UK) and collagen IV (Abcam, UK), both at a dilution of 1:200 in phosphate-buffered saline (PBS). The sections were subsequently incubated with a secondary goat anti-mouse IgG (H + L) HRP and a secondary goat anti-rabbit IgG (H + L) HRP at 37 °C for 30 min. Sections were consequently stained with diaminobenzidine for 10 min, counterstained with hematoxylin (Beyotime, China), covered with coverslips, and observed under an optical microscope.

For the immunofluorescence staining, tissue sections were blocked with 3% bovine serum albumin (BSA) in PBS for 30 min at 25 °C. Sections were incubated overnight at 4 °C with antibodies against p66Shc (phosphor S36) (Abcam, UK), Sirt1 (Cell Signaling Technology, USA), alpha-smooth muscle actin (α-SMA) (Abcam, UK), collagen IA1 (Proteintech, USA), and CTGF (Abcam, UK) at a dilution of 1:100. All secondary antibodies were diluted (1:2000) and incubated at 25 °C for 2 h.

Primary cells were fixed in 4% paraformaldehyde for 30 min at room temperature and then permeabilized with 0.3% Triton X-100. After washing with PBS for three times, cells were blocked with 3% BSA for 30 min at 25 °C. Cells were incubated overnight at 4 °C with antibodies against follicle-stimulating hormone receptor (FSHR) (Abcam, UK), luteinizing hormone receptor (LHR) (Cell Signaling Technology, USA), p66Shc (Servicebio, China), p66Shc (phosphor S36) (Abcam, UK), Sirt1 (Cell Signaling Technology, USA), collagen IA1 (Proteintech, USA), CTGF (Abcam, UK) and α-SMA (Abcam, UK), at a dilution of 1:100. After washing with PBS for three times, cells were incubated at 25 °C for 2 h with secondary antibodies. Nuclei were counterstained with 4′,6-diamidino-2-phenylindole (DAPI) at a dilution of 1:2000 for 30 min and photographed using an Olympus laser scanning confocal microscope (FV3000). Images were quantified with Image-Pro Plus 6.0.

### Measurement of intracellular ROS production

Cells were plated onto glass-bottom tissue culture dishes. Following treatment with DHT, resveratrol and siRNA, the primary cells were loaded with dichloro-dihydro-fluorescein diacetate (DCF-DA, 10 µM) and incubated for 30 min at 37 °C. Medium was discarded and cells were washed with PBS for three times on ice. Images of the cells were captured using an Olympus laser scanning confocal microscope (FV3000). Images were analyzed by Image-Pro Plus 6.0.

### Determination of mitochondrial membrane potential

Mitochondrial were visualized with the MitoTracker Red CMXRos dye (Yeasen, China). Briefly, primary GCs were were plated on coverslips and incubated with 200 nM MitoTracker Red CMXRos at 37 °C for 30 min. Mitochondrial membrane potential was determined by the JC-1 probe (Beyotime, China) according to the supplier’s instructions. The red and green JC-1 fluorescence ratio was calculated. Images were quantified with Image-Pro Plus 6.0.

### Western blot

Ovarian proteins were extracted by RIPA lysis buffer (Beyotime, China) containing 1 mM Pierce™ Phosphatase Inhibitor (Thermo Fisher Scientific, USA) and 0.1% Halt™ Protease Inhibitor Cocktail (Thermo Fisher Scientific, USA). Equal amounts of total proteins were separated by 10% sodium dodecyl sulfate–polyacrylamide gel electrophoresis and the protein bands were then transferred onto polyvinylidene difluoride membranes (Merck Millipore, USA). Target bands were incubated with corresponding primary antibodies against p66Shc (phosphor S36) (1:500, Abcam, UK), Sirt1 (1:1000, Cell Signaling Technology, USA), TGF-β (1:1000, Cell Signaling Technology, USA), CTGF (1:1000, Abcam, UK), β-catenin (1:1000, Cell Signaling Technology, USA), α-SMA (1:1000, Abcam, UK), AR (1:1000, Abcam, UK), p-p53 (1:1000, Abcam, UK) and GAPDH (1:5000, Bioworld Technology, China) at 4 °C, overnight, followed by the addition of HRP-labeled secondary antibodies. The blots were visualized using chemiluminescent detection (Merck Millipore, Germany). Densitometric analysis was performed with Image J.

### Quantitative real-time PCR (qRT-PCR)

Total RNA was extracted from ovaries, GCs with TRIzol reagent (Beyotime, China) and cDNA was synthesized with a reverse transcription kit (Vazyme, China). Quantitative RT-PCR was performed with the ABI Viia 7 Real-Time PCR system (ABI, USA) by using the SYBR Green PCR Master Mix (Vazyme, China) and the primers are shown in Table 1. The critical threshold cycle (Ct) value was determined for each reaction, which was transformed into relative quantification data using the 2^−∆∆Ct^ method. The housekeeping gene β-actin was used as an internal control.

**Table 1:** Primers of the genes used in the study

β-actin F	TTCCTTCCTGGGTATGGAAT
β-actin R	GAGGAGCAATGATCTTGATC
p66Shc F	CTGAAGGTGTGGTTCGGACA
p66Shc R	ACTGCCTGCAGAGATGATGG
Sirt1 F	CCAGATCCTCAAGCCATGT
Sirt1 R	TTGGATTCCTGCAACCTG
TGF-β F	TACTGCTTCAGCTCCACAGAGA
TGF-β R	CAGACAGAAGTTGGCATGGTAG
α-SMA F	AGGGACTAATGGTTGGAATGG
α-SMA R	CAATCTCACGCTCACGCTCGGCAGTAG
CTGF F	CATTAAGAAGGGCAA A AAGTGC
CTGF R	CACACCCCACAGAACTTAGCC
AR F	TCTGGTTGTCACTACG GAGC
AR R	TGCAATCATTTCTGCTGGCAC

### Statistical analysis

All statistical analyses were performed with GraphPad (Prism 7.00). Statistical method for multiple comparisons was implemented by one- or two-way ANOVA software followed by Tukey’s post hoc test. Binary variables were compared using *t* test. All p-values less than 0.05 were considered significant.

## Results

### Hyperandrogenic ovarian dysfunction and fibrosis are improved by treatment with resveratrol possibly via the suppression of OS

**Fig. 1:**
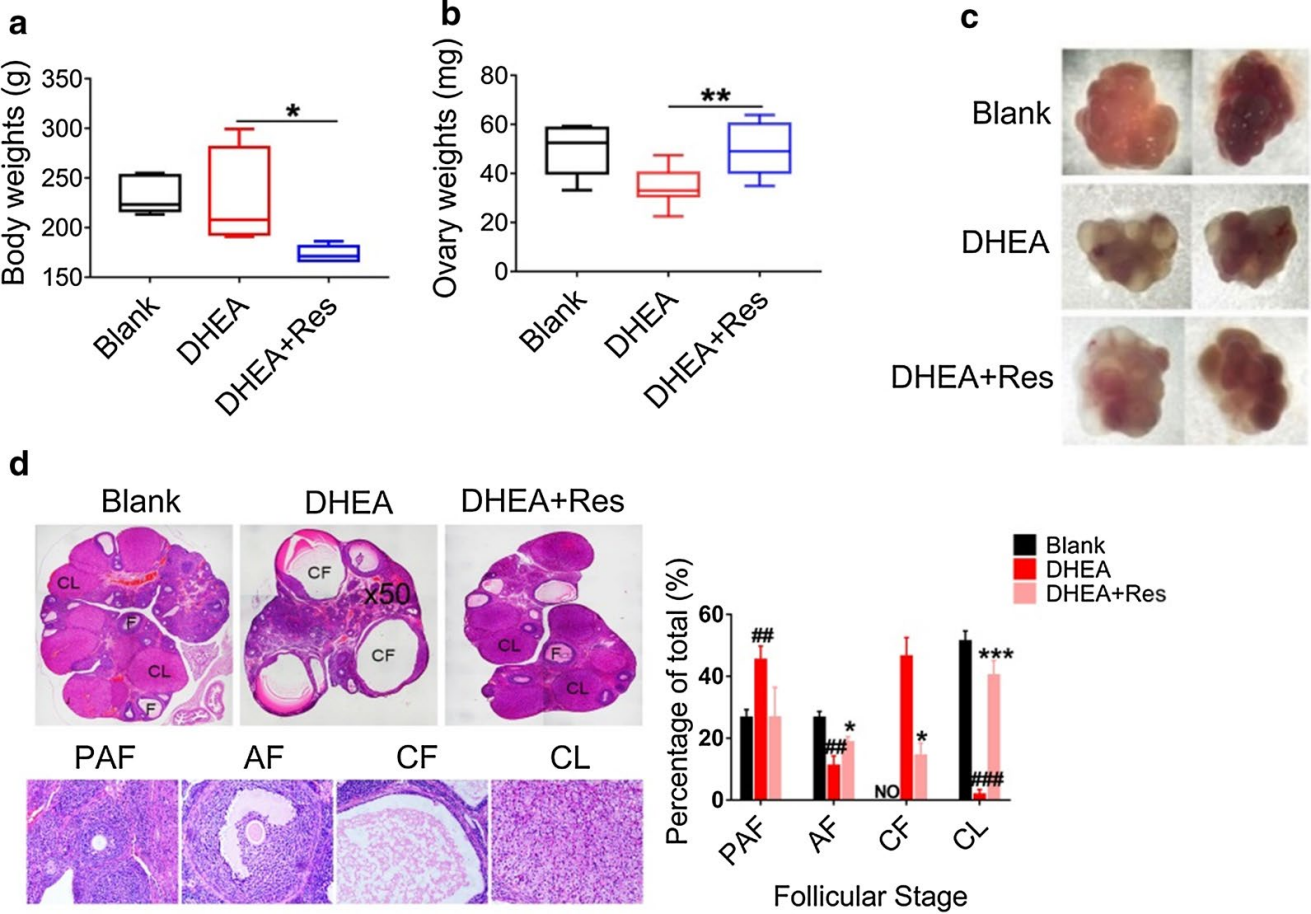
Ovarian morphology is improved after treatment with resveratrol in dehydroepiandrosterone-exposed rats. Rats received DHEA for induction of polycystic ovarian syndrome together with or without resveratrol treatment. **a** Rat body weights were measured on the day of sacrifice (day 36). **b** Average weight of both ovaries was measured. **c** Photographs of the morphology of the ovaries from each treatment group were shown. **d** Ovarian and follicular morphology was assessed by H&E staining (5×). The percentage of each follicle was shown on the right. n = 7 in each group. Three independent experiments were performed with similar results. Data are shown as the mean ± SD. ^##^p ≤ 0.01, ^###^p ≤ 0.001 vs. Blank; *p ≤ 0.05, **p ≤ 0.01, ***p ≤ 0.001 vs. DHEA treatment. DHEA, dehydroepiandrosterone; Res, resveratrol; PAF, preantral and early antral follicle; AF, antral follicle; CF, cystic follicles; CL, corpus luteum

DHEA-induced PCOS rats that had been treated with resveratrol demonstrated lower body weights compared with untreated PCOS rats (Fig. 1a). The ovaries of hyperandrogenic PCOS rats were dramatically smaller, which was markedly improved upon treatment with resveratrol (Fig. 1b). In addition, androgen-induced thick fibrotic capsules and high numbers of multiple immature follicles were substantially reduced after resveratrol treatment, whereas the numbers of luteal cells and antral follicles were increased evidently (Fig. 1c, d). Resveratrol has been traditionally used to activate Sirt1 in various tissues [32, 33]. Sirt1 can partially decrease the expression of p66Shc and exert anti-OS activities. Therefore, our data may suggest that the stimulation of Sirt1 may improve ovarian morphology and function in PCOS rats.

**Fig. 2:**
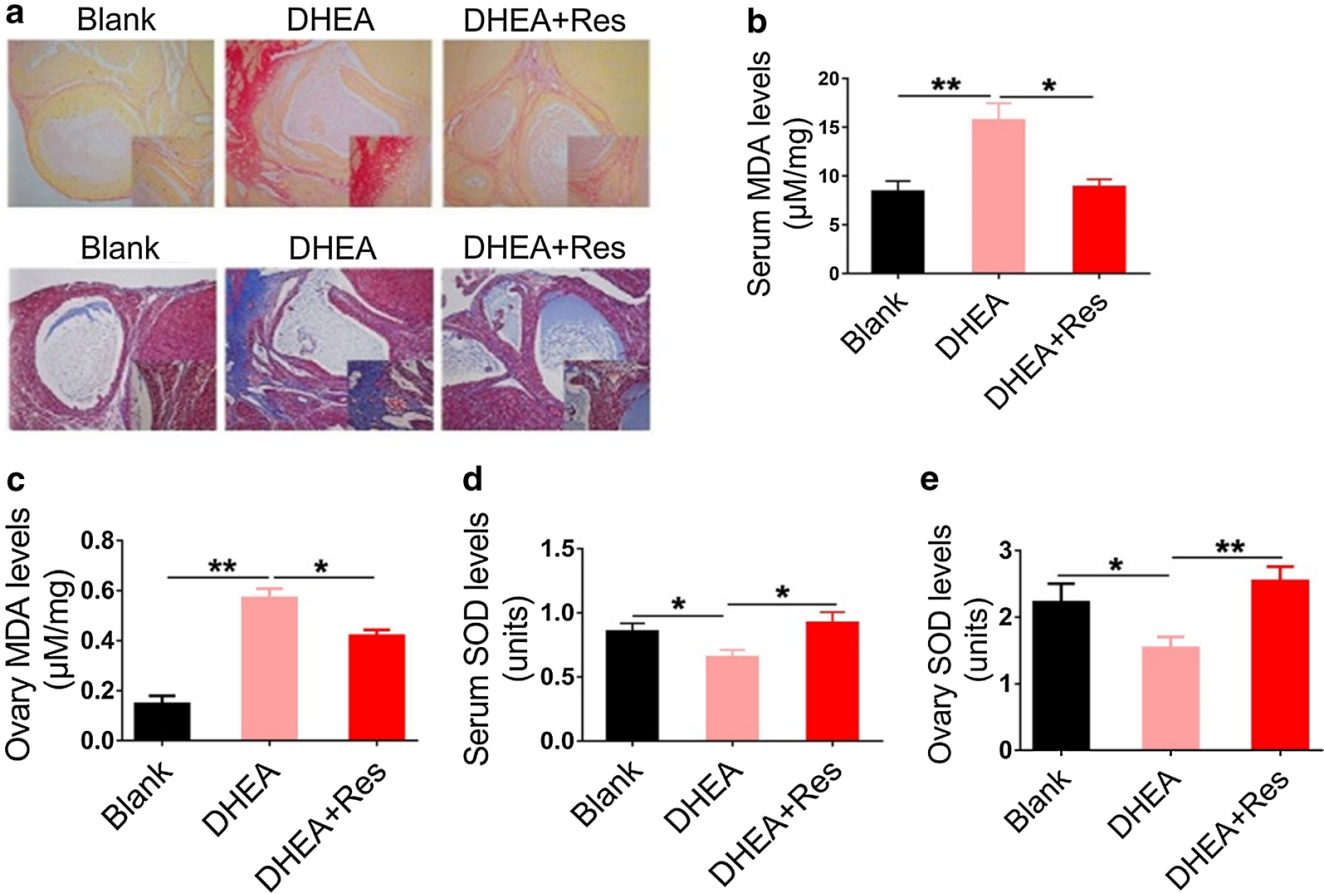
Resveratrol suppresses DHEA-induced ovarian fibrosis and oxidative stress. **a** Collagen in ovarian slices was revealed by Sirius Red and Masson staining (10×). Images are representative of three independent experiments with similar results. Serum (**b**) and ovarian (**c**) malondialdehyde (MDA) levels were analyzed using an enzymatic colorimetric method. Serum (**d**) and ovarian (**e**) superoxide dismutase (SOD) activity was analyzed using an enzymatic colorimetric method. n = 7 in each group. Three independent experiments were performed with similar results. Data are shown as the mean ± SEM. *p ≤ 0.05, **p ≤ 0.01. DHEA, dehydroepiandrosterone

We used Sirius Red and Masson staining, a connective tissue stain specific for collagen I and III fibers, to evaluate ovarian fibrosis [34]. Ovarian interstitial fibrosis was inhibited in the presence of resveratrol which was revealed by Sirius Red and Matson staining (Fig. 2a). Collagen IV also plays an important role in the progression of fibrosis. Our data demonstrated that collagen IV was mainly expressed in ovarian stroma and follicular membranes. Compared with the PCOS group, collagen IV was substantially inhibited after treatment with resveratrol (Additional file 1: Fig. S1A).

The OS protein p66Shc can be suppressed by activating Sirt1 [35]. Therefore, we undertook to investigate whether inhibiting the expression of p66Shc by resveratrol could suppress ovarian OS, thereby restraining further fibrosis progression. To determine the levels of OS in PCOS rats, we measured the levels of MDA and SOD in serum and ovaries. Serum and ovarian levels of MDA were markedly decreased and the levels of SOD were profoundly enhanced after treatment with resveratrol (Fig. 2b–e).

### Treatment with resveratrol inhibits p66Shc phosphorylation and fibrogenic factors in vivo

**Fig. 3:**
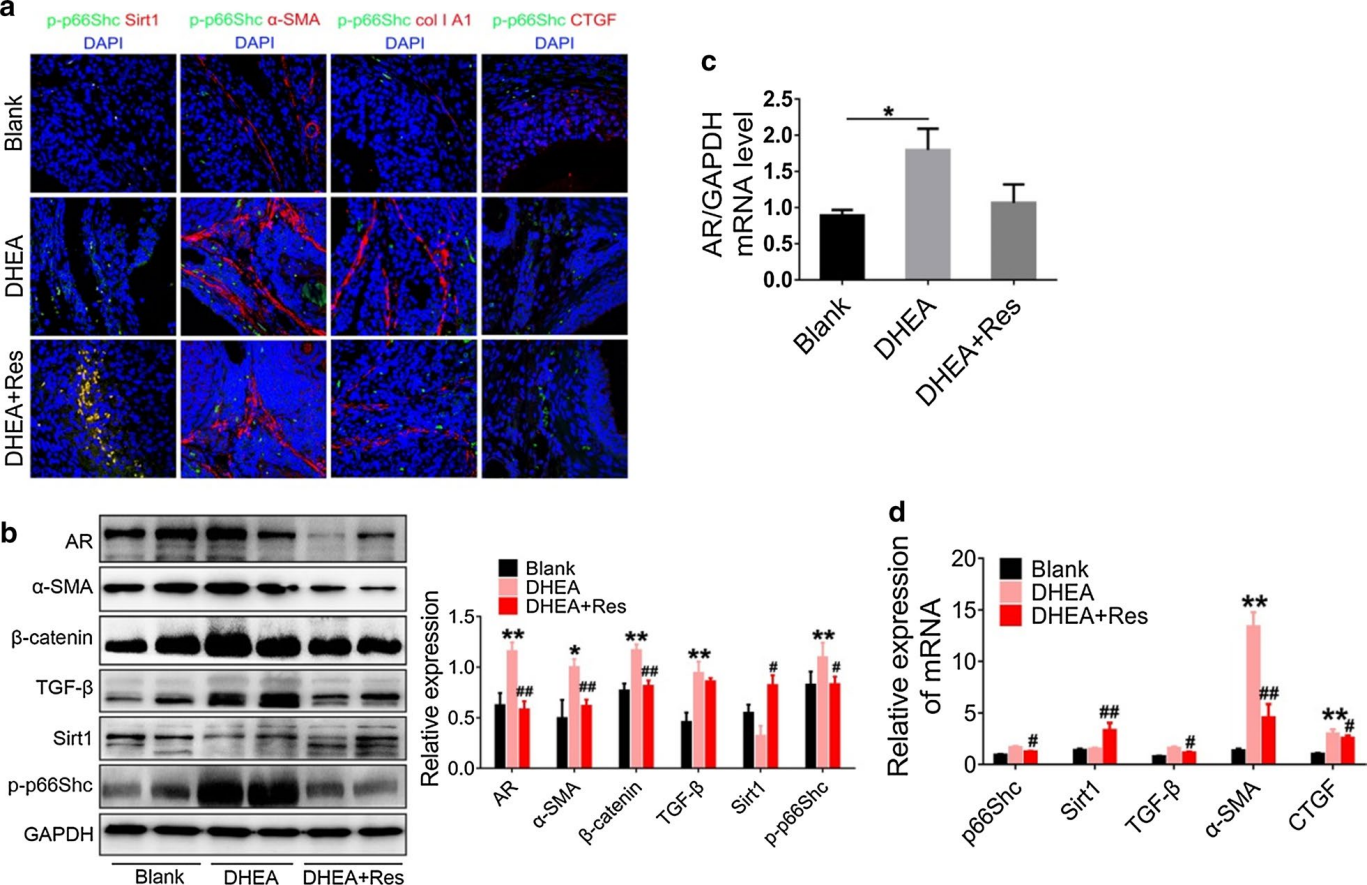
Resveratrol inhibits p66Shc phosphorylation and fibrotic factors activation in rat ovaries. Rats received DHEA for the induction of polycystic ovarian syndrome, together with or without resveratrol treatment. **a** Sirt1, α-SMA, collagen I A1, CTGF, and p-p66Shc expression in rat ovaries (60×) was analyzed by double immunofluorescence staining. Images are representative of three independent experiments with similar results. **b** The expression of fibrotic factors and p-p66Shc in ovaries was assessed by western blot. The panel on the right shows the quantitative analysis. **c** The mRNA levels of AR was analyzed by real-time PCR. **d** Relative expression of p66Shc and fibrotic factors in rat ovaries was determined by real-time PCR. n = 7 in each group. Three independent experiments were performed with similar results. Data are shown as mean ± SD & mean ± SEM. *p ≤ 0.05, **p ≤ 0.01 vs. Blank; ^#^p ≤ 0.05, ^##^p ≤ 0.01 vs. DHEA treatment. *DHEA* dehydroepiandrosterone, *AR* androgen receptor, *CTGF* connective tissue growth factor, *Sirt1* sirtuin 1, *p-p66Shc* phosphorylated 66-kDa Src homology 2 domain-containing protein

Treatment with resveratrol substantially enhanced the Sirt1 protein expression, which was mainly located in the cytoplasm of GCs, accompanied with the down-regulation of phosphorylateion of p66Shc (p-p66Shc) (Fig. 3a and Additional file 1: Fig. S1B). In addition, the levels of α-SMA, collagen IA1, CTGF, and collagen IV were all significantly decreased in the rats treated with resveratrol (Fig. 3a and Additional file 1: Fig. S1A). In order to further confirm that resveratrol could suppress phosphorylation of p66Shc and ovarian fibrosis, we next analyzed protein expression levels of various fibrotic factors using immunoblotting. P-p66Shc and the profibrotic factors, including TGF-β, β-catenin and α-SMA, were markedly decreased in resveratrol-treated rats (Fig. 3b). In addition, the levels of AR were also reduced (Fig. 3c). Consistently, the mRNA levels of AR, p66Shc, and the fibrogenic factors, including TGF-β, α-SMA, and CTGF, were also decreased. In contrast, the expression of Sirt1 mRNA was substantially upregulated upon treatment with resveratrol (Fig. 3d).

### DHT promotes p66Shc phosphorylation resulting in mitochondrial dysfunction

**Fig. 4:**
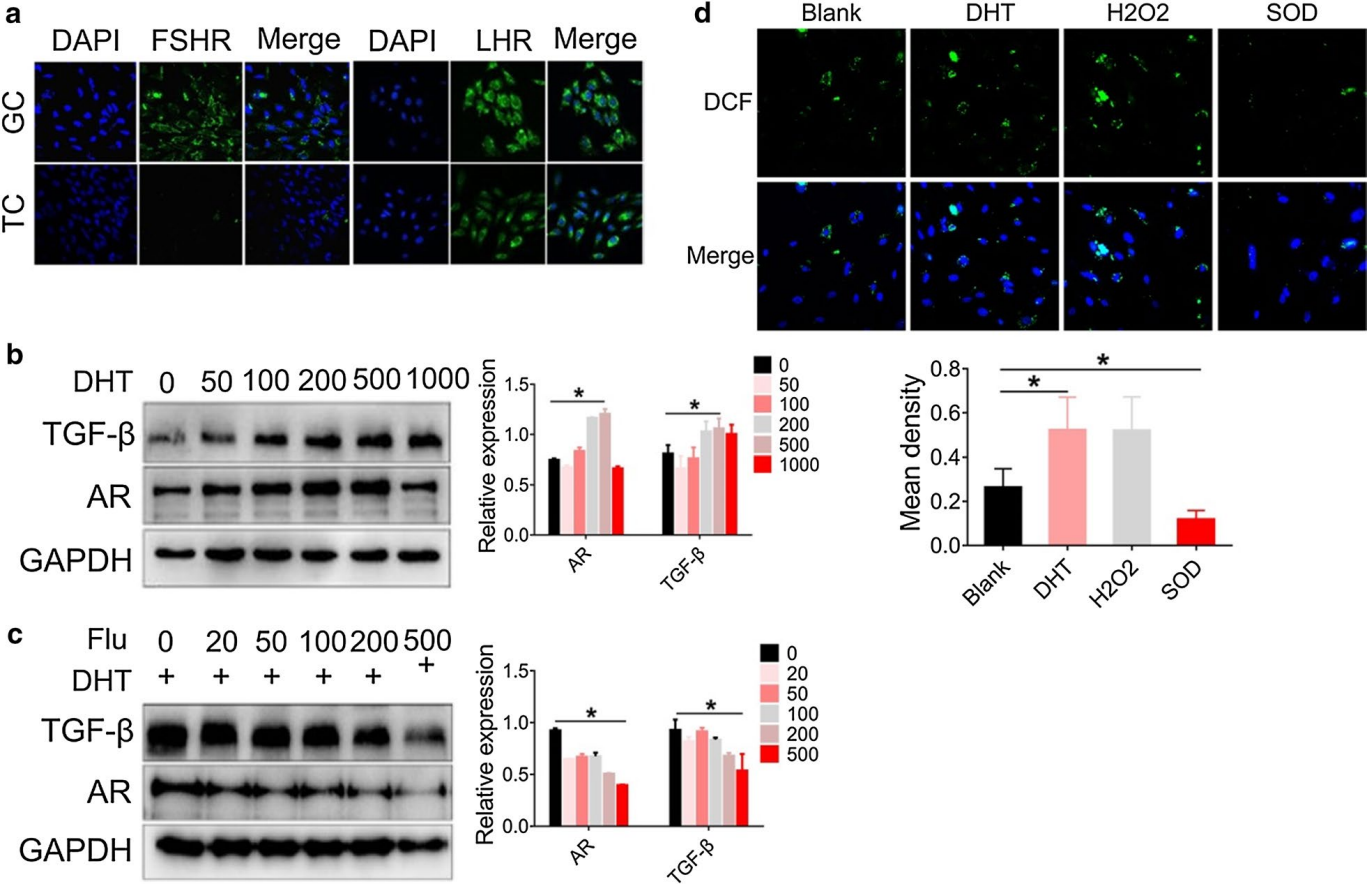
Dihydrotestosterone promotes generation of ROS and TGF-β in GCs. **a** The expression of FSHR and LHR in GCs and TCs was measured by immunofluorescence staining (Alexa Fluor 488) and the nuclei were stained with DAPI (40×). Images are representative of three independent experiments with similar results. **b** GCs treated with various concentrations of DHT for 24 h followed by immunoblot analysis of the AR and TGF-β proteins. **c** GCs were pre-treated with DHT (500 nM) for 24 h, followed by treatment with flutamide (Flu) at various concentrations (0, 20, 50, 100, 200, 500 μM) for 24 h. The AR and TGF-β protein levels were measured by western blot. **d** ROS generation in GCs following various treatments was measured using the DCF-DA probe. DCF-DA fluorescence (green fluorescence) was measured by confocal microscopy (40×). Images are representative of three independent experiments with similar results. Quantification of the fluorescence is shown. Three independent experiments were performed with similar results. Data are shown as the mean ± SD. *p ≤ 0.05. *DHT* dihydrotestosterone, *AR* androgen receptor, *TGF-β* transforming growth factor-beta, *FSHR* follicle-stimulating hormone receptor, *LHR* luteinizing hormone receptor, *GCs* granulosa cells, *TCs* theca cells, *ROS* reactive oxygen species

Follicle-stimulating hormone (FSH) and luteinizing hormone (LH) promote the development of follicles, resulting in the production of large amounts of estradiol (E2) [36]. Androgens synthesized by TCs enter the GCs through vasculature, followed by conversion to E2. FSHR is considered as the marker of GCs [32]. Consistently, our results show that FSHR was highly expressed in GCs, but not in TCs. In addition, LHR was expressed in both GCs and TCs at various levels (Fig. 4a). By measuring FSHR, we could roughly estimate the cell purity of GCs. Furthermore, we treated cells with DHT at various concentrations and extracted the protein for further analysis. Our data confirmed that the expression of AR and TGF-β in GCs was dose-dependently increased by DHT and the expression reached its peak at a DHT concentration of 500 nM (Fig. 4b). Upon treatment with the antagonist of DHT, flutamide, the expression of AR and TGF-β in GCs was dose-dependently decreased (Fig. 4c). Consistently, immunoblot analysis of GCs confirmed that TGF-β and p66Shc/p-p66Shc were markedly increased after treatment with DHT for at least 24 h (Additional file 2: Fig. S2). Our results demonstrated that increased fibrosis was indeed associated with the hyperandrogenic conditions.

Hydrogen peroxide (H_2_O_2_) is commonly used in models of OS-induced apoptosis [37]. In this study, we treated GCs with 500 μM H_2_O_2_ to induce OS as a positive control [38, 39]. Furthermore, treatment with DHT in GCs increased ROS levels according to analysis by the DCF-DA probe. DHT-induced increase in ROS levels was consistent with H_2_O_2_ treatment (Fig. 4d). In addition, SOD treatment reduced ROS levels (Fig. 4d). These findings suggest that increased ROS levels in GCs were indeed associated with hyperandrogenism.

**Fig. 5:**
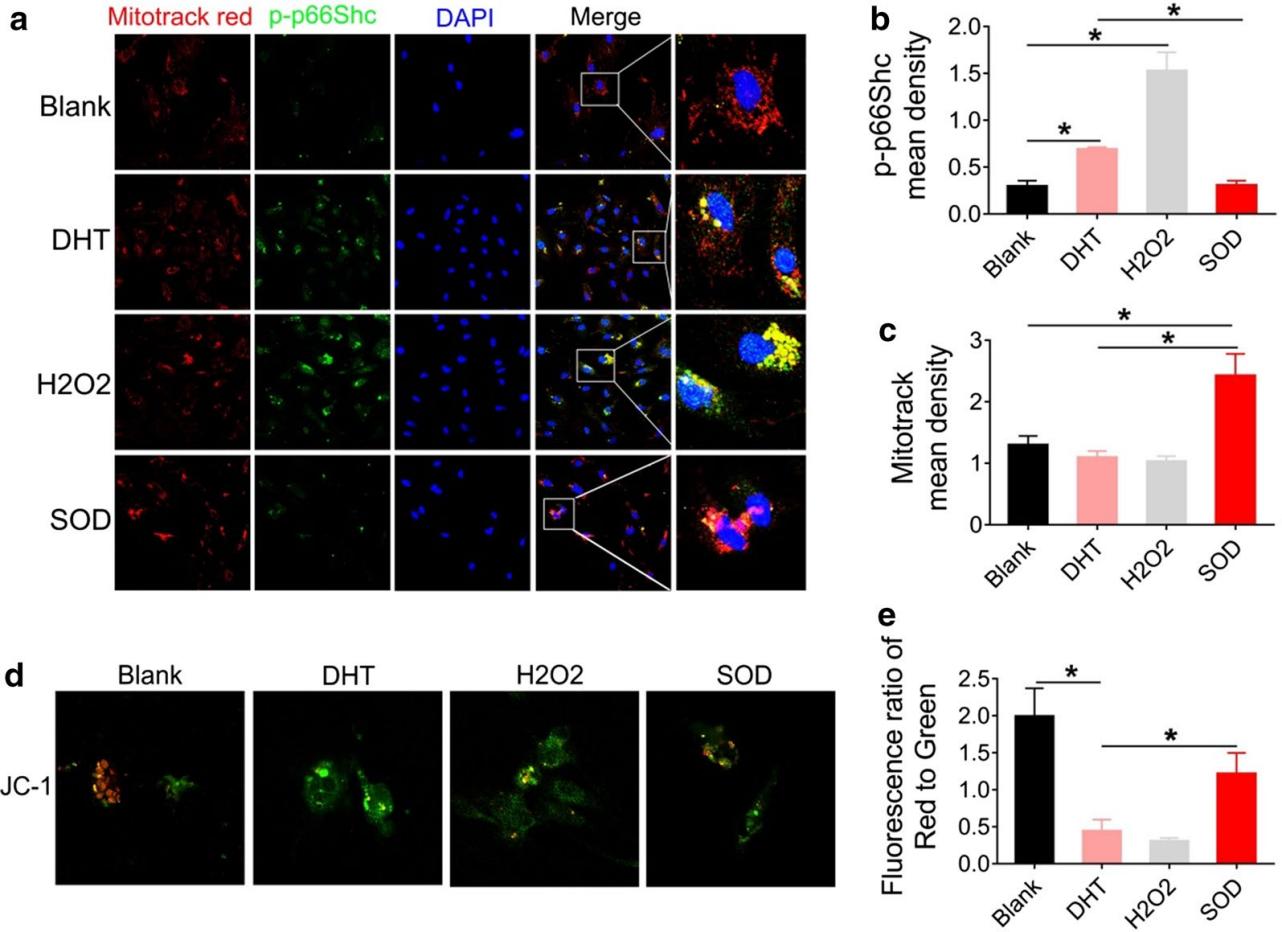
Dihydrotestosterone promotes p66Shc phosphorylation resulting in mitochondrial dysfunction. Granulosa cells from naïve rats were treated with DHT, H_2_O_2_ or SOD. **a** p-p66Shc expression (Alexa Fluor 488) and mitochondria (MitoTracker Red) were revealed by confocal microscopy. **b** Quantification of the p-p66Shc fluorescence intensity is shown. **c** Quantification of MitoTrack Red intensity is shown. **d** Mitochondrial membrane potential was analyzed by the ratio of JC-1 monomers/polymers (60×). **e** Quantitative analysis of the ratios of the red to green fluorescence. Images are representative of three independent experiments with similar results. Data are shown as the mean ± SEM. *p ≤ 0.05. DHT, dihydrotestosterone; H_2_O_2_, hydrogen peroxide; SOD, superoxide dismutase; p-p66Shc, phosphorylated 66-kDa Src homology 2 domain-containing protein

Mitochondria are major sites of superoxide radical generation, which can detoxify ROS. However, excess ROS generation can destroy these defense mechanisms and lead to mitochondrial damage. Therefore, we analyzed whether H_2_O_2_-induced ROS is involved in p66Shc phosphorylation and mitochondrial dysfunction. GCs were treated with 500 μM H_2_O_2_ followed by immunofluorescence staining. Our data showed that p-p66Shc expression was restricted to mitochondria and H_2_O_2_-mediated upregulation was observed, which was consistent with DHT treatment (Fig. 5a–c). However, p-p66Shc expression was prominently declined in SOD-treated GCs (Fig. 5a, b), and the visualization and quantification of MitoTracker Red were markedly increased in SOD-treated GCs (Fig. 5a, c). The dysfunction of mitochondrial membrane potential, measured by JC-1 monomers, was induced by treatment with H_2_O_2_ or DHT. In contrast, mitochondrial membrane potential remained higher in blank controls and SOD treatment groups (Fig. 5d, e). Taken together, these data suggest that p66Shc promoted the expression of fibrotic factors, possibly as a result of DHT-induced oxidative stress and mitochondrial dysfunction in GCs.

### p66Shc silencing attenuates hyperandrogen-induced oxidative stress resulting in the inhibition of the expression of fibrotic factors in vitro

**Fig. 6:**
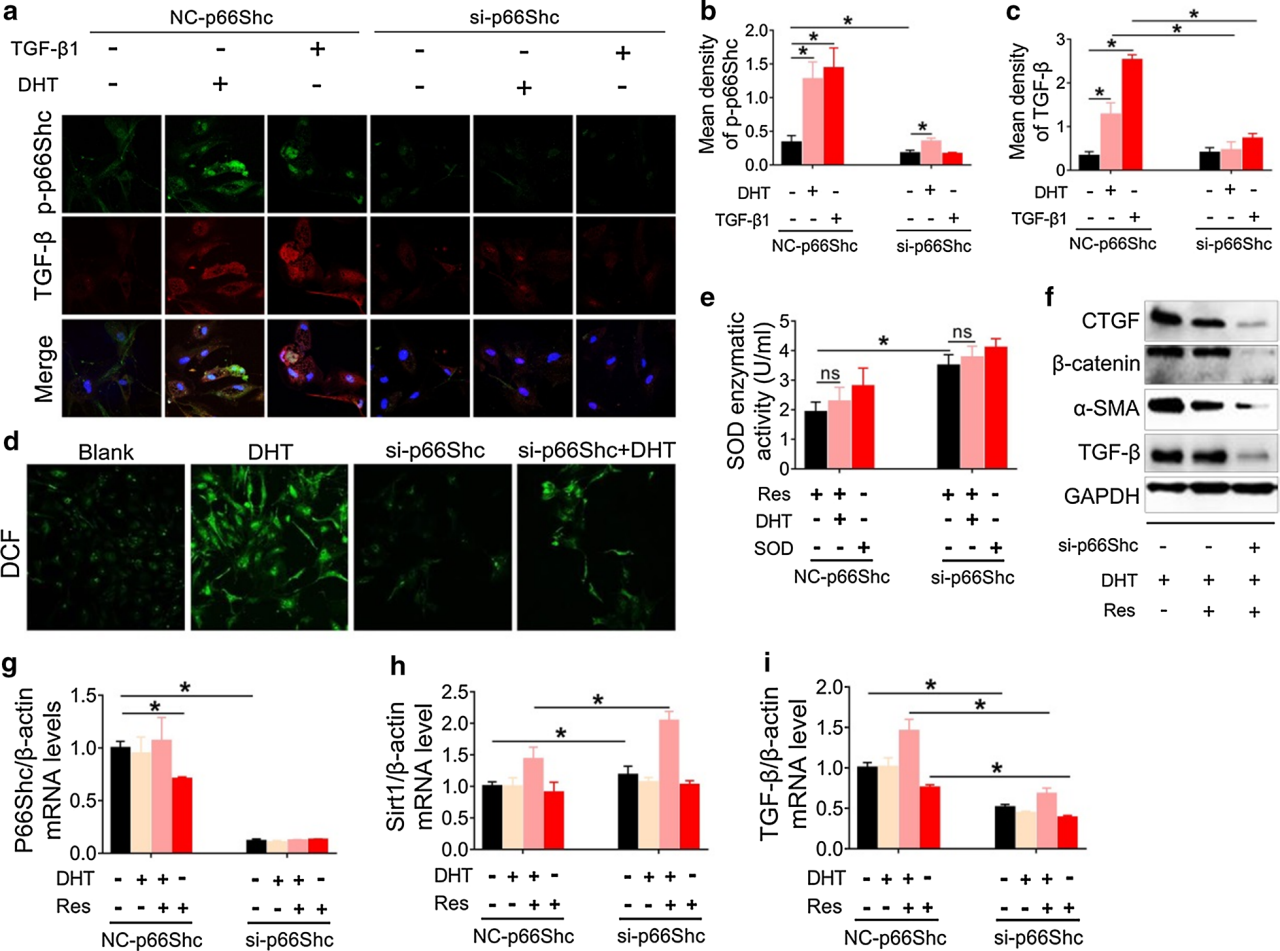
p66Shc silencing suppresses oxidative stress and fibrotic factor activation in granulosa cells. Granulosa cells were transfected with p66Shc siRNA followed by treatment with DHT, TGF-β1 or resveratrol. **a** Levels of p-p66Shc (Alexa Fluor 488) and TGF-β (Cy3) were measured with immunofluorescence (60×). Images are representative of three independent experiments with similar results. Quantification of the p-p66Shc (**b**) and TGF-β (**c**) fluorescence is shown. **d** ROS was assessed by the DCF-DA probe using confocal microscopy (40×). Images are representative of three independent experiments with similar results. **e** SOD enzymatic activity in granulosa cells was measured by colourimetric method. **f** The expression of fibrotic factors and AR was assessed by western blot assay. mRNA expression of p66Shc (**g**), Sirt1 (**h**), and TGF-β (**i**) was analyzed by real-time PCR. Three independent experiments were performed with similar results. Data are shown as the mean ± SD. *p ≤ 0.05. *DHT* dihydrotestosterone, *TGF-β* transforming growth factor-beta, *p-p66Shc* phosphorylated 66-kDa Src homology 2 domain-containing protein, *ROS* reactive oxygen species, *SOD* superoxide dismutase, *Sirt1* sirtuin 1, *AR* androgen receptor

OS is one of the major causes of idiopathic infertility, spontaneous abortion, and impaired implantation in preeclampsia [40]. Our data suggested that phosphorylation of p66Shc was augmented in GCs after treatment with DHT. We thus tried to examine the role of p66Shc in OS and found that it subsequently contributed to the activation of fibrotic factors. p66Shc knockdown by siRNA inhibited the potential of DHT- or TGF-β1-induced enhancement of p-p66Shc and TGF-β in GCs isolated from the rat ovary confirmed by immunofluorescence staining (Fig. 6a–c). Double immunofluorescence staining showed that p-p66Shc was localized in TGF-β-positive cells, and the increased expression of p-p66Shc and TGF-β induced by DHT or TGF-β1 was eliminated by p66Shc silencing (Fig. 6a). Furthermore, ROS in GCs was analyzed using the DCF-DA probe. Results showed that the enhancement of ROS stimulated by DHT was significantly inhibited by p66Shc silencing (Fig. 6d). Conversely, p66Shc silencing markedly increased the enzymatic activity of SOD in GCs (Fig. 6e). However, DHT modestly increased the enzymatic activity after treatment with resveratrol irrespective of p66Shc silencing (Fig. 6e). p66Shc silencing dramatically enhanced the suppressive effect of resveratrol on the expression of some fibrotic factors, including TGF-β, α-SMA, β-catenin, and CTGF, as confirmed by western blot (Fig. 6f). Furthermore, we also measured the mRNA levels of p66Shc, Sirt1, and TGF-β after p66Shc silencing. The results showed that Sirt1 mRNA expression was markedly enhanced with p66Shc knockdown by siRNA. In contrast, p66Shc and TGF-β mRNA levels were significantly decreased after p66Shc silencing (Fig. 6g–i). However, the mRNA expression of p66Shc and TGF-β was markedly increased after treatment with both DHT and resveratrol irrespective of p66Shc silencing (Fig. 6g, i). Taken together, our findings suggested that p66Shc silencing by siRNA enhanced the inhibitory effect on DHT-induced enhancement of fibrotic factors.

### p66Shc contributes to fibrotic factor activation by suppressing mitochondrial function

**Fig. 7:**
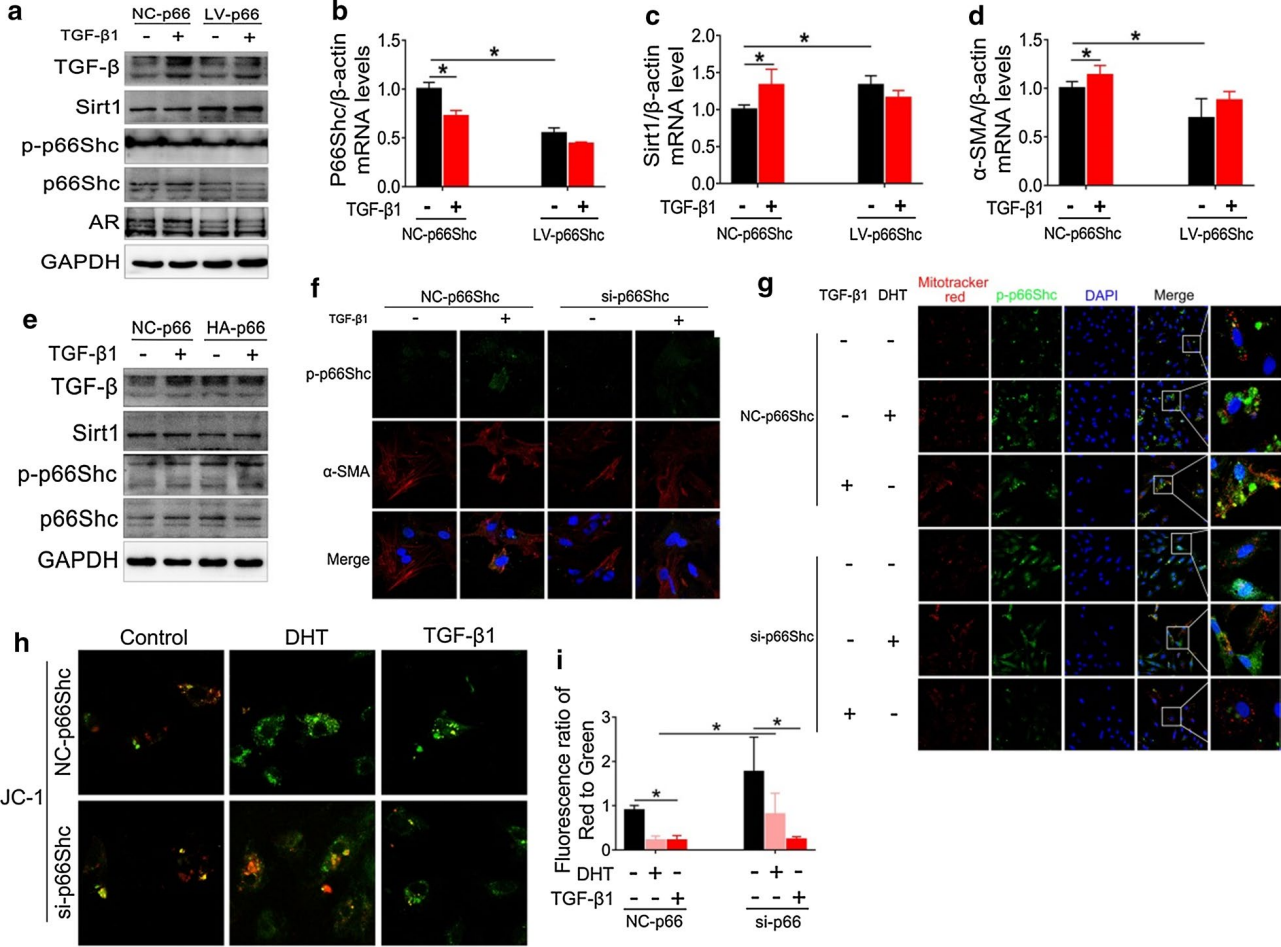
p66Shc silencing inhibits fibrotic factors activation and improves mitochondrial dysfunction. p66Shc in granulosa cells was silenced by lentivirus or overexpressed by a plasmid vector. After 72 h, the cells were then treated with DHT or TGF-β1. **a** The expression of p66Shc, p-p66Shc, Sirt1, TGF-β and AR was analyzed by western blot assay. mRNA levels of p66Shc (**b**), Sirt1 (**c**), and α-SMA (**d**) was analyzed by real-time PCR. **e** The expression of p66Shc, p-p66Shc, Sirt1, and TGF-β was assessed by western blot assay. **f** The expression of p-p66Shc (Alexa Fluor 488) and α-SMA (Cy3) were analyzed using immunofluorescence staining (60×). Images are representative of three independent experiments with similar results. **g** p-p66Shc (Alexa Fluor 488) and mitochondria were revealed by immunofluorescence and MitoTracker Red staining, respectively using confocal microscopy. **h** Mitochondrial membrane potential was analyzed by JC-1 monomers/polymers (60×). Images are representative of three independent experiments with similar results. **i** Quantitative analysis of the ratios of the ratios red to green fluorescence is shown. Three independent experiments were performed with similar results. Data are shown as the mean ± SD. *p ≤ 0.05. DHT, dihydrotestosterone; TGF-β1, transforming growth factor-beta 1; p-p66Shc, phosphorylated 66-kDa Src homology 2 domain-containing protein; Sirt1, sirtuin 1; AR, androgen receptor; α-SMA, alpha-smooth muscle actin; GCs, granulosa cells

TGF-β is implicated as a key factor in tissue fibrosis [8]. To confirm whether p66Shc was involved in the activation of fibrotic factors, we transfected GCs with lentivirus-p66Shc (Fig. 7a–d) or plasmid-p66Shc (Fig. 7e) followed by TGF-β1 challenge. p66Shc silencing substantially suppressed TGF-β1-mediated expression of p66Shc/p-p66Shc and TGF-β at the protein levels, as well as p66Shc and α-SMA at the mRNA levels (Fig. 7a, b, d). As expected, p66Shc silencing evidently promoted TGF-β1-mediated suppression of Sirt1 expression at both protein and mRNA levels (Fig. 7a, c). Taken together, these data supported that, following silencing of p66Shc, TGF-β1 failed to efficiently stimulate fibrotic factor activation. In contrast, p66Shc overexpression dramatically amplified TGF-β1-induced expression of p-p66Shc and TGF-β (Fig. 7e). However, the expression of Sirt1 was only modestly decreased with p66Shc overexpression (Fig. 7e). Additionally, p66Shc overexpression upregulated TGF-β protein expression, although no further enhancement could be induced by the addition of TGF-β1 (Fig. 7e). These western blot and real-time PCR data were also confirmed by immunofluorescence staining. Increase in p-p66Shc and α-SMA expression induced by TGF-β1was eliminated by the transduction with lentivirus-p66Shc (Fig. 7f). In order to confirm the correlation of p-p66Shc and mitochondrial dysfunction, we tried to locate the distribution of p-p66Shc expression in mitochondria using immunofluorescence staining. We observed co-localization of p-p66Shc and mitochondria revealed by MitoTracker Red. TGF-β1- or DHT-induced expression of p-p66Shc in mitochondria was suppressed by lentivirus-mediated p66Shc-silencing (Fig. 7g). Furthermore, mitochondrial membrane potential collapse, examined by JC-1 staining, as a result of TGF-β1 or DHT treatment was improved after p66Shc silencing (Fig. 7h, i). These data indicate that the phosphorylation of p66Shc is the key factor responsible for DHT-induced fibrotic factor activation, which is possibly mediated by mitochondrial dysfunction.

### Sirt1 suppresses fibrotic factor activation in a p66Shc-dependent manner

**Fig. 8:**
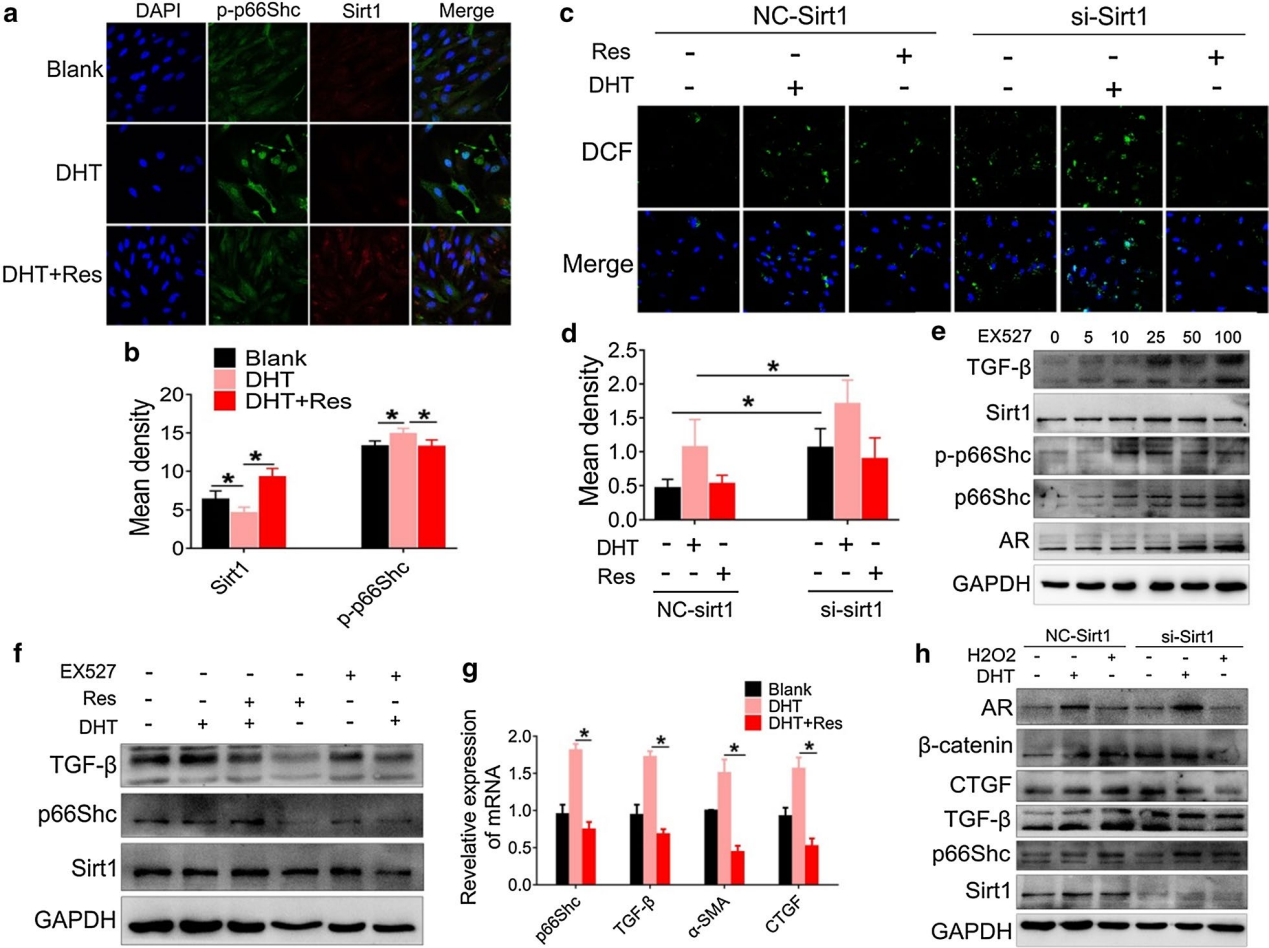
Sirt1 suppresses fibrotic factors activation in a p66Shc-dependent manner. Sirt1 in granulosa cells was silenced using siRNA. After 48 h, the cells were treated with DHT, resveratrol, H_2_O_2_, or EX527. **a** p-p66Shc and Sirt1 were analyzed by double immunofluorescence staining (40×). Images are representative of three independent experiments with similar results. **b** Quantification of the p-p66Shc and Sirt1 fluorescence is shown. **c** ROS was measured using a DCF-DA probe, and assessed by confocal microscopy (40×). Images are representative of three independent experiments with similar results. **d** Quantification of DCF fluorescence is shown. **e** Granulosa cells were treated with various concentrations of EX527. The expression of p66Shc, p-p66Shc, Sirt1, TGF-β, and AR was analyzed by western blot assay. **f** The expression of p66Shc, Sirt1 and TGF-β was assessed by western blot assay in granulosa cells after treatment with DHT, resveratrol, or EX527. **g** mRNA levels of p66Shc, TGF-β, α-SMA, and CTGF were measured by real-time PCR. **h** The expression of p66Shc, Sirt1, TGF-β, CTGF, β-catenin, and AR in granulosa cells treated with DHT or H_2_O_2_ was analyzed by western blot after Sirt1 silencing. Three independent experiments were performed with similar results. Data are shown as the mean ± SD. *p ≤ 0.05. *DHT* dihydrotestosterone, *TGF-β* transforming growth factor-beta, *p-p66Shc* phosphorylated 66-kDa Src homology 2 domain-containing protein, *Sirt1* sirtuin 1, *AR* androgen receptor, *α-SMA* alpha-smooth muscle actin, *CTGF* connective tissue growth factor, *GCs* granulosa cells, *DCF* dichlorodihydrofluorescein, *ROS* reactive oxygen species, EX527, a Sirt1 inhibitor

The antagonistic effects of p66Shc and Sirt1 in the activation of OS raised the possibility that Sirt1 may inhibit p66Shc to suppress fibrotic factor activation. To confirm whether Sirt1 suppressed fibrotic factor activation in a p66Shc-dependent manner, we measured the expression of p-p66Shc and Sirt1 in GCs after treatment with DHT or resveratrol, using double immunofluorescence staining. Our data demonstrated that DHT-induced expression of p-p66Shc was suppressed by resveratrol, an agonist of Sirt1 (Fig. 8a, b). Furthermore, GCs were treated with DHT or resveratrol following transfection with Sirt1 siRNA and ROS was measured using the DCF-DA probe. Our data showed that Sirt1 silencing markedly enhanced the expression of SOD levels irrespective of the treatment with either DHT or resveratrol (Fig. 8c, d). Additionally, we treated GCs with various concentrations of EX527 (MCE, China, HY-15452), an inhibitor of Sirt1 enzymatic activity. The levels of p66Shc, p-p66Shc, TGF-β, and AR were dose-dependently increased (Fig. 8e). These results suggested that Sirt1 was possibly involved in AR suppression. Furthermore, we treated GCs with DHT, resveratrol or EX527. Resveratrol could downregulate the protein expression of TGF-β and p66Shc, whereas EX527 demonstrated the opposite effects (Fig. 8f). However, Sirt1 was only modestly regulated by these treatments (Fig. 8f). We also confirmed that resveratrol could decrease the expression of p66Shc and the fibrotic factors, including TGF-β, α-SMA, and CTGF, at the mRNA level (Fig. 8g). Furthermore, Sirt1 silencing increased the protein expression of TGF-β, CTGF, and β-catenin, as well as AR and p66Shc (Fig. 8h). However, oxidative stress induced by H_2_O_2_ failed to exert any influence on the activation of fibrotic factors after knocking down Sirt1 (Fig. 8h). Taken together, our data support that Sirt1 was able to suppress the activation of fibrotic factor by inhibiting the expression of p66Shc.

## Discussion

A previous study has demonstrated that resveratrol can inhibit fibrosis by regulating ROS [41]. Furthermore, the pro-fibrotic factor TGF-β could stimulate ROS production, which in turn activates fibrogenic factors such as TGF-β and CTGF [42]. In the present study, we demonstrated that ROS could be inhibited by inhibition of p66Shc production, through the activation of Sirt1 with resveratrol or p66Shc knockdown.

Androgen-induced PCOS rats are confirmed to demonstrate ovarian hyperfibrosis [7]. Despite the overall complexity involved in the molecular mechanisms, it is very likely that OS may contribute to ovarian fibrosis. Our data showed that the serum and ovarian MDA levels were increased while the SOD levels were decreased in DHEA-treated rats. OS can cause both the peroxidation of cell membrane-associated unsaturated lipids and the oxidation of proteins, DNA and steroid components leading to further impairment in cellular integrity and functionality [43]. A previous study indicated that p66Shc, an isoform of the ShcA adapter molecule, modulates intracellular redox balance by increasing the ROS concentration as a critical mediator of intracellular oxidative signal transduction [23]. Moreover, p66Shc has been known to be negatively regulated by Sirt1 through deacetylation of histone H3 lysine 9, and binding to the p66Shc promoter region [26]. In this study, administration of resveratrol, the activator of Sirt1, resulted in a markedly decrease in p66Shc and fibrotic factors. It is widely recognized that ROS can alter p66Shc activity, leading to activation of pro-fibrotic factors and deposition of collagen. Despite the growing body of evidence implicating the critical role of p66Shc in the pathophysiology of metabolic diseases, there is limited information about the mechanisms that negatively regulate p66Shc expression.

Sirt1 has been extensively studied and has been shown to affect metabolism, aging, and tolerance to OS through its ability to deacetylate transcription factors, co-regulators, histones, etc. [24]. Sirt1-mediated p66Shc reduction is associated with the prevention of OS-mediated endothelial dysfunction and senescence, as well as the pathophysiological process of PCOS. Sirt1 also exerts a significant regulatory function in energy metabolism in response to nutrient stimulating signals, whereas p66Shc is currently regarded as a principal regulator of overnutrition-mediated insulin resistance. In support of these observations, we found that rats with long-term exposure to DHEA had significantly decreased the expression of Sirt1 in the ovary. Interestingly, resveratrol could enhance the expression of Sirt1, indicating that resveratrol might be able to protect rats from DHEA-induced ovarian injury by upregulating Sirt1. In DHT-exposed cells, resveratrol pretreatment attenuated the expression of p66Shc and TGF-β, whereas the inhibition of Sirt1 by EX527 or si-Sirt1 increased their expression, indicating that Sirt1 provides protection against androgen-induced ovarian injury. EX527 exerts an inhibitory effect on Sirt1 activity without affecting Sirt1 expression on both mRNA and protein levels.

Further analysis suggests that p-p66Shc was predominantly expressed in GCs, the main location for sex hormone synthesis. TCs secrete androgen, which penetrates the basement membrane into the follicle and acts as a synthetic E2 substrate [44]. FSH is capable of stimulating CYP19 activation, followed by conversion of testosterone and androstenedione into estradiol and estrone aromatase in GCs, which is a major rate-limiting stage in E2 synthesis [45]. Our data also revealed that serum total testosterone levels were not down-regulated with the treatment of resveratrol (Additional file 3: Fig. S3). However, the expression of androgen receptor was markedly decreased. This suggests that the observed protective effect of p66Shc on androgen-induced ovarian fibrosis was likely not associated with downregulation of androgen. However, the exact mechanism leading to this change in sex hormone production is still unclear.

The interaction between GCs and oocytes is the main regulatory mechanism of follicular development. Oocytes, encapsulated by GCs throughout the developmental stages, play a multifunctional regulatory role in follicular development and atresia [46]. In the present study, thinner ovarian granular cell layers were observed in rats with PCOS. In addition, expression of p-p53 protein in the ovaries of these rats was upregulated (Additional file 4: Fig. S4). There is some evidence indicating that both p53 and p66Shc play essential roles in promoting OS in the vascular system, and p53 can promote the expression of p66Shc [26, 47]. These findings suggest that p53 may play an important role in hyperandrogen-induced ovarian OS, which is mediated by p66Shc.

In this study, ROS was found to be substantially increased after treatment with DHT in GCs. Downregulation of p66Shc substantially reduced ROS generation and the expression of fibrogenic factors in follicles. We observed that the phosphorylation of p66Shc was mainly expressed in the mitochondria of GCs, especially following androgen overexpression, resulting in the destruction of the mitochondrial membrane potential. Taken together, our data suggest that hyper-androgenism could upregulate p66Shc-induced ROS production in PCOS rats, and the enhanced ROS in turn caused mitochondrial dysfunction, further leading to increased transcription of the fibrogenic factors and collagen deposition. However, in our study, DHT only modestly increased SOD enzymatic activity after treatment with resveratrol irrespective of p66Shc silencing. It supports that resveratrol may promote DHT to induce secretion of SOD in GCs, which is possibly independent of p66Shc. Thus, it is still unclear whether p66Shc is the major regulator of ovarian OS, and the precise mechanisms of hyperandrogen-induced ovarian fibrosis have yet to be fully revealed.

Notwithstanding, we have provided compelling evidence herein that p66Shc-mediated OS plays a substantial role in hyperandrogen-induced ovarian fibrosis. This research may shed light on novel pathways and molecules that can be exploited for future development of therapeutic strategies for PCOS.

## Conclusions

In summary, p66Shc plays an essential role in hyperandrogen-induced ovarian OS and fibrosis. Resveratrol has a suppressive effect on p66Shc through activating Sirt1. Moreover, suppression of p66Shc improves hyperandrogen-induced ovarian OS and fibrosis.

## Supplementary information

**Additional file 1: Figure S1.** Resveratrol significantly downregulates the expression of collagen IV and p-p66Shc in vivo.

**Additional file 2: Figure S2.** DHT promoted the activation of p66Shc TGF-β.

**Additional file 3: Figure S3.** Serum hormone levels are not subject to change under resveratrol treatment.

**Additional file 4: Figure S4.** Expression of the p-p53 protein is significantly increased after treatment with dehydroepiandrosterone.

## Data Availability

All data generated or analyzed during this study are included in this published article.

## Abbreviations

PCOS: Polycystic ovary syndrome
OS: Oxidative stress
GCs: Granulosa cells
TCs: Theca cells
DHEA: Dehydroepiandrosterone
DHT: Dihydrotestosterone
AR: Androgen receptor
ROS: Reactive oxygen species
TGF-β: Transforming growth factor-β
CTGF: Connective tissue growth factor
α-SMA: α Smooth muscle actin
RNS: Reactive nitrogen species
p-p66Shc: Phosphorylated 66-kDa Src homology 2 domain-containing protein
Sirt1: Sirtuin type 1
Res: Resveratrol
MDA: Malondialdehyde
SOD: Superoxide dismutase
col IA1: Collagen IA1
col IV: Collagen IV
siRNA: Small interfering RNA
H_2_O_2_: Hydrogen peroxide
PAF: Preantral and early antral follicle
AF: Antral follicle
CF: Cystic follicles
CL: Corpus luteum
FSHR: Follicle-stimulating hormone receptor
LHR: Luteinizing hormone receptor
CYP19: Cytochrome p450 19
