# Supplementary material for: Suppression of p66Shc prevents hyperandrogenism-induced ovarian oxidative stress and fibrosis
Source: J Transl Med. 2020 Feb 17;18:84. doi: 10.1186/s12967-020-02249-4 (PMC7027222; doi:10.1186/s12967-020-02249-4)
Supplement: Supplementary file 1 — Additional file 1: Figure S1. Resveratrol significantly downregulates the expression of collagen IV and p-p66Shc in vivo. [file 12967_2020_2249_MOESM1_ESM.docx]

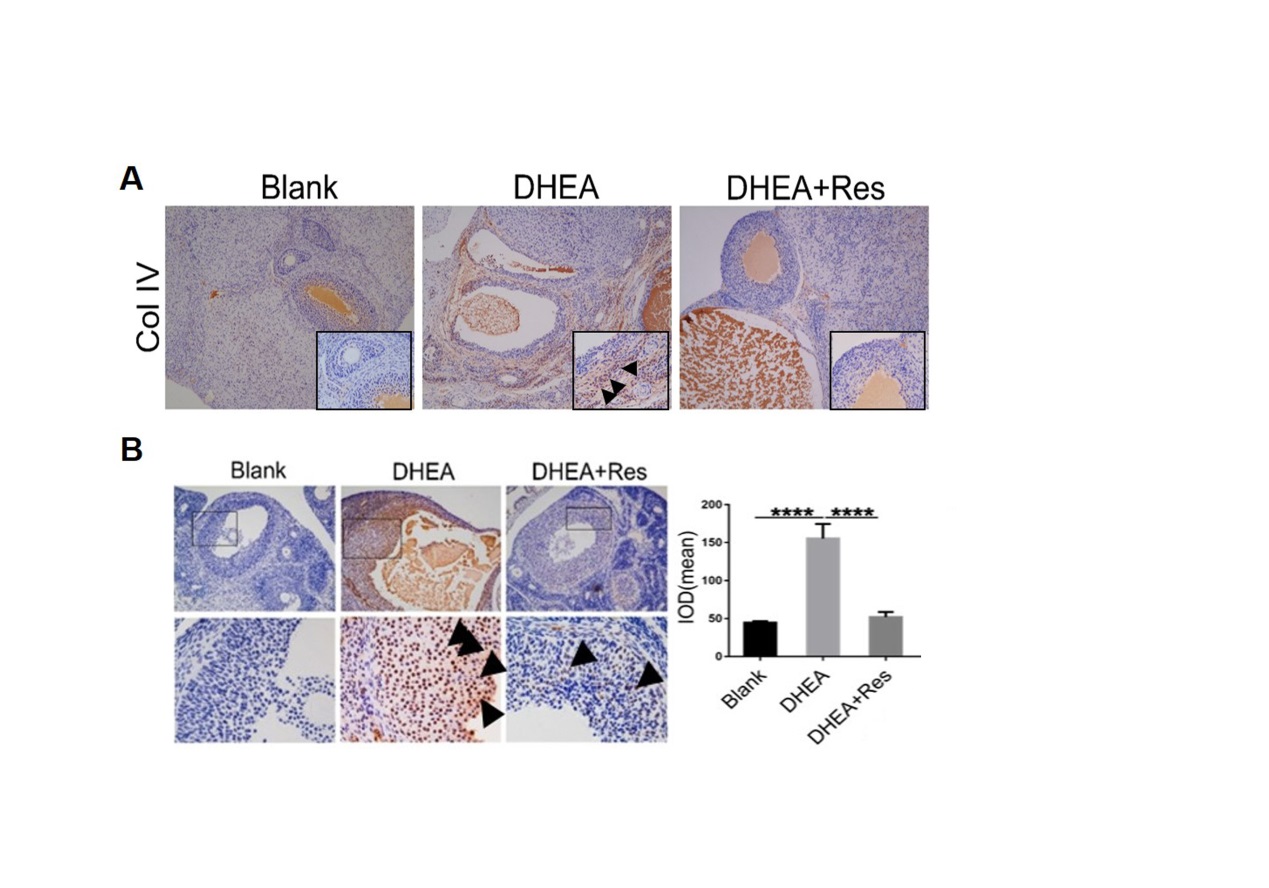


**Figure S1. Resveratrol significantly downregulates the expression of collagen IV and p-p66Shc in vivo.** Rats received dehydroepiandrosterone for the induction of polycystic ovarian syndrome, together with or without resveratrol treatment. (A to B) Collagen IV protein (A) (10X) and p66Shc (phosphor S36) protein (B) (10X) and (40X) in the rat ovary were assessed by immunohistochemistry. The panel on the right shows the quantification of the signal. Data are shown as the mean ± SD. ****p ≤ 0.0001. Res, resveratrol.
