# Supplementary material for: Suppression of p66Shc prevents hyperandrogenism-induced ovarian oxidative stress and fibrosis
Source: J Transl Med. 2020 Feb 17;18:84. doi: 10.1186/s12967-020-02249-4 (PMC7027222; doi:10.1186/s12967-020-02249-4)
Supplement: Supplementary file 2 — Additional file 2: Figure S2. DHT promoted the activation of p66Shc TGF-β. [file 12967_2020_2249_MOESM2_ESM.docx]

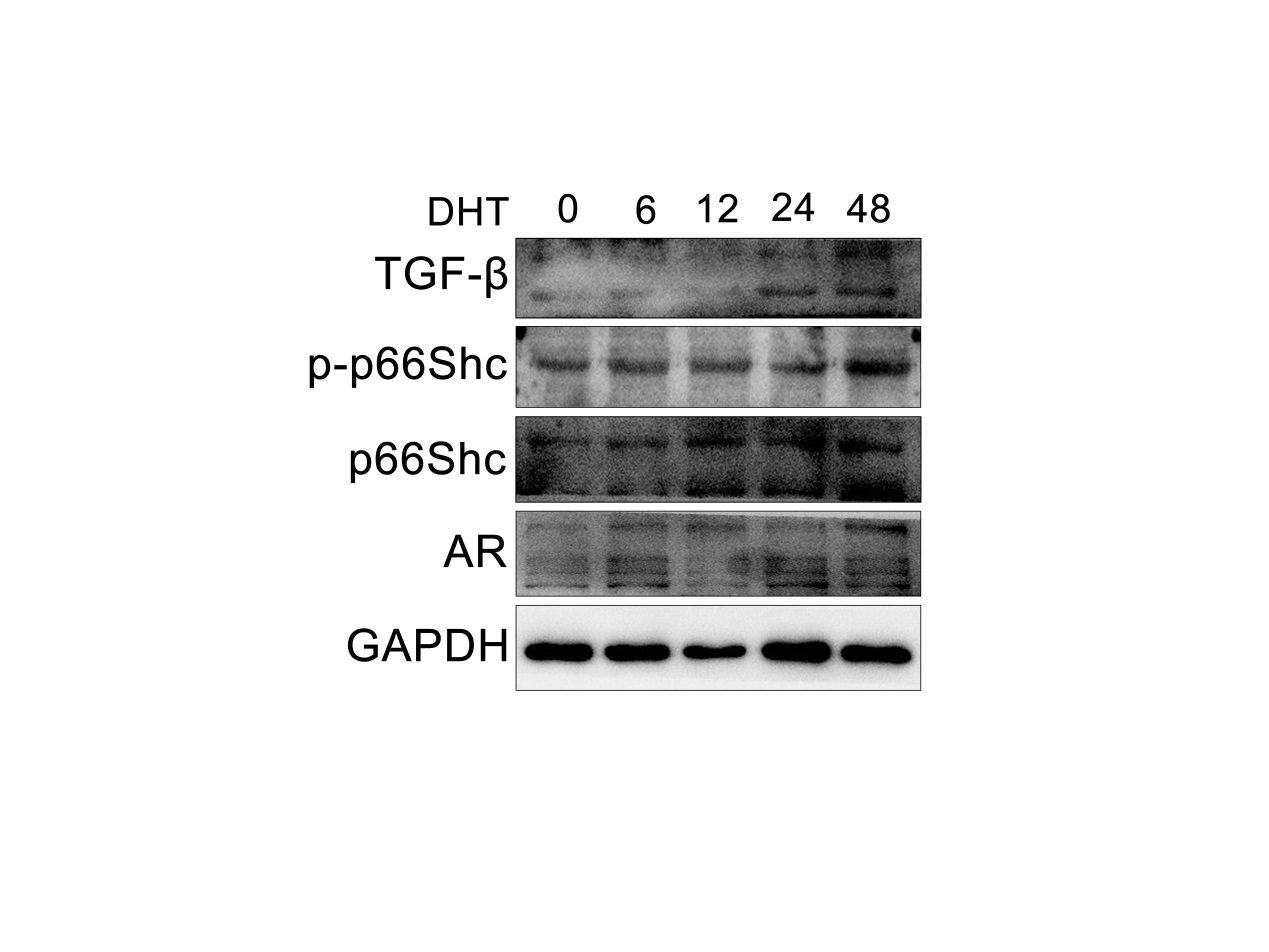


**Figure S2. DHT promoted the activation of p66Shc TGF-β.** Granulosa cells were treated with 500 nM dihydrotestosterone for 0, 6, 12, 24, 48 h. The expression of p66Shc, p-p66Shc, TGF-β, and AR was assessed by western blot assay. TGF-β, transforming growth factor-beta; p-p66Shc, phosphorylated 66-kDa Src homology 2 domain-containing protein; AR, androgen receptor.
