# Supplementary material for: Suppression of p66Shc prevents hyperandrogenism-induced ovarian oxidative stress and fibrosis
Source: J Transl Med. 2020 Feb 17;18:84. doi: 10.1186/s12967-020-02249-4 (PMC7027222; doi:10.1186/s12967-020-02249-4)
Supplement: Supplementary file 3 — Additional file 3: Figure S3. Serum hormone levels are not subject to change under resveratrol treatment. [file 12967_2020_2249_MOESM3_ESM.docx]

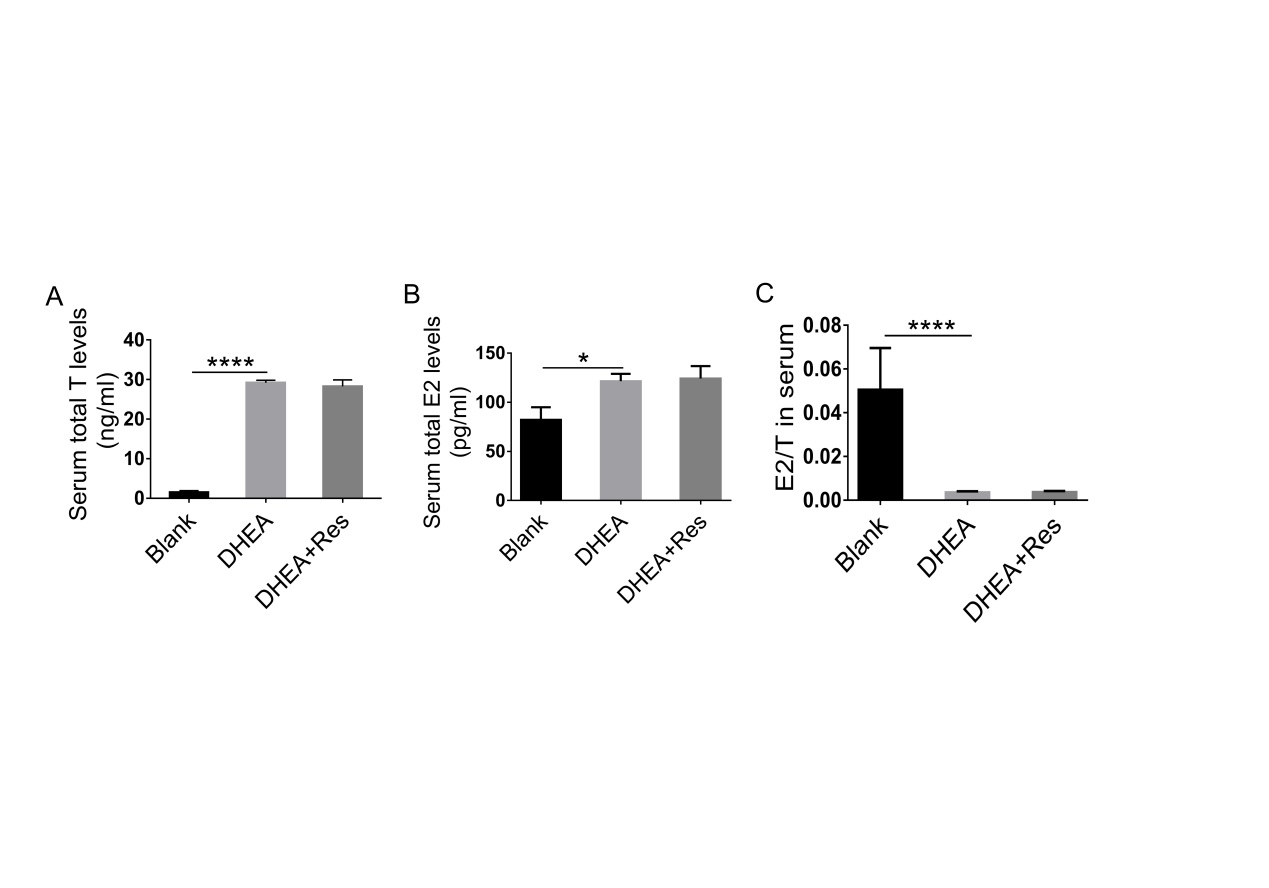


**Figure S3. Serum hormone levels are not subject to change under resveratrol treatment.** (A to B) Serum total testosterone (T) (A) and estradiol (E2) (B) levels were measured with ELISA. (C) E2/T levels are indicated. n=7 in each group. Three independent experiments were performed with similar results. Data are shown as the mean ± SD. *p ≤ 0.05, ****p ≤ 0.0001.
