# Supplementary material for: Suppression of p66Shc prevents hyperandrogenism-induced ovarian oxidative stress and fibrosis
Source: J Transl Med. 2020 Feb 17;18:84. doi: 10.1186/s12967-020-02249-4 (PMC7027222; doi:10.1186/s12967-020-02249-4)
Supplement: Supplementary file 4 — Additional file 4: Figure S4. Expression of the p-p53 protein is significantly increased after treatment with dehydroepiandrosterone. [file 12967_2020_2249_MOESM4_ESM.docx]

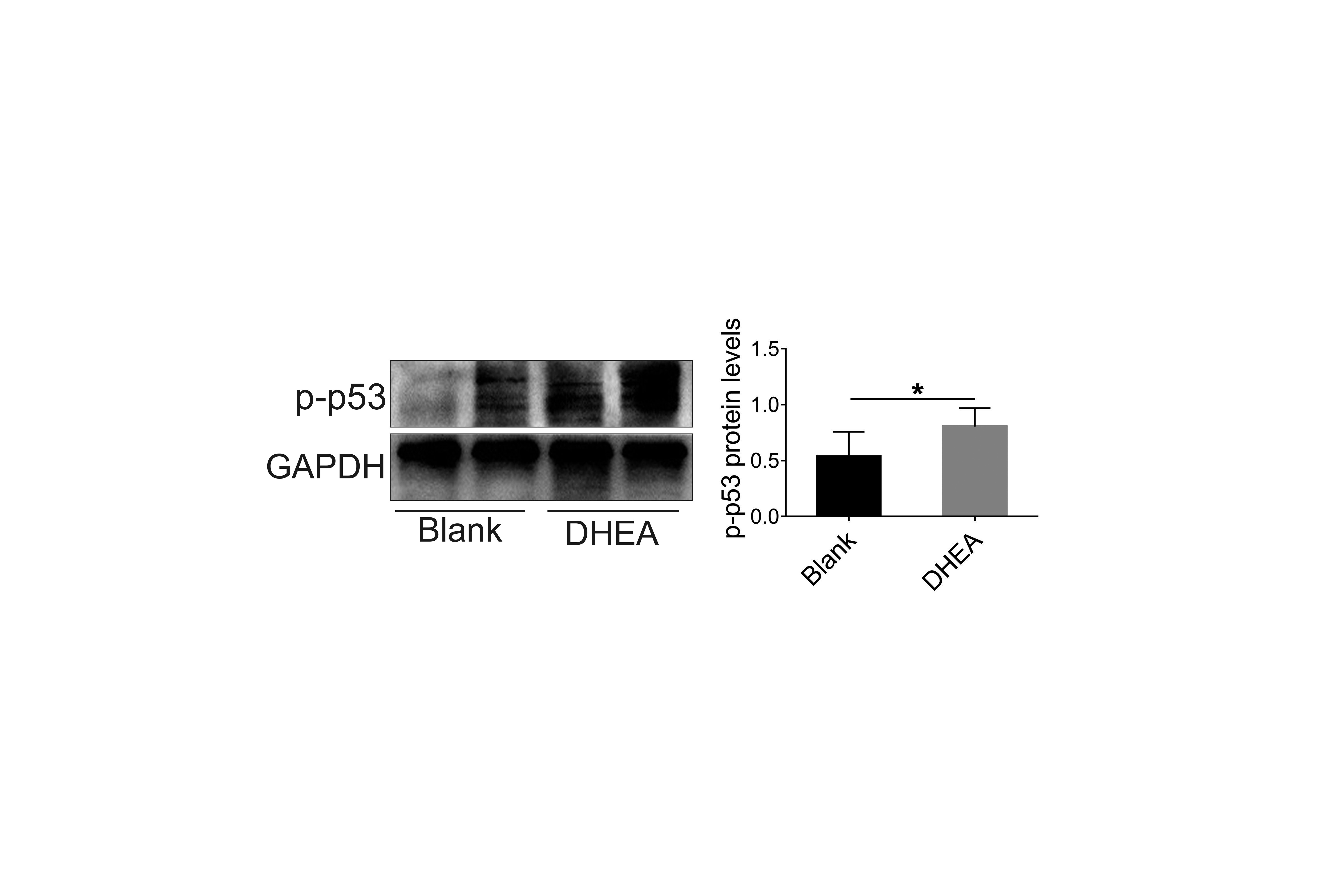


**Figure S4. Expression of the p-p53 protein is significantly increased after treatment with dehydroepiandrosterone.** Rats received dehydroepiandrosterone for the induction of polycystic ovarian syndrome. The p-p53 protein in the rat ovary was measured by western blot assay; the quantification of p-p53 is shown. Three independent experiments were performed with similar results. Data are shown as the mean ± SD. *p ≤ 0.05.
